# Efficacy of acupuncture on upper limb dysfunction after stroke: a randomized controlled trial protocol with surface electromyography evaluation

**DOI:** 10.3389/fmed.2025.1615762

**Published:** 2025-07-10

**Authors:** Liying Wang, Zining Guo, Jiewen Zhang, Run Lin, Xiaorong Tang, Cui Gao, Shaoyang Cui, Nenggui Xu

**Affiliations:** ^1^Shenzhen Hospital (Fu Tian) of Guangzhou University of Chinese Medicine, Shenzhen, China; ^2^South China Research Center for Acupuncture and Moxibustion, Medical College of Acu-Moxi and Rehabilitation, Guangzhou University of Chinese Medicine, Guangzhou, China

**Keywords:** acupuncture, upper limb dysfunction, randomized controlled trial, stroke, neuromuscular effect

## Abstract

**Background:**

Upper extremity functional reconstruction remains a major clinical challenge in post-stroke neurorehabilitation. Acupuncture has unique advantages as a complementary alternative therapy. Thus, in this study, we aim to compare sham acupuncture to reveal the efficacy and safety of acupuncture in the treatment of upper limb dysfunction post-stroke with surface electromyography (sEMG), a validated objective assessment tool, and a subjective index.

**Methods:**

In this prospective, principal-investigator-blinded randomized controlled trial, 74 patients who meet the inclusion criteria are randomly assigned to the acupuncture and sham acupuncture groups in a 1:1 ratio using a central randomization system. The patients receive the same routine basic treatment. The core acupoints selected in the acupuncture group are GV20 (Bai hui), DU14 (Da zhui), and LI11 (Qu chi). Six non-meridian acupuncture points are selected for the sham acupuncture group. The treatment cycle lasts for 4 weeks, five times per week, for 20 treatments. The Fugl–Meyer Upper Limb Assessment scale score is used as the main outcome. Secondary outcomes include the Motor Status Scale, Action Research Arm Test, Motor Assessment Scale, Self-rating Anxiety Scale, and Self-rating Depression Scale scores. Simultaneously using sEMG as an auxiliary efficacy indicator.

**Conclusion:**

This study assesses the effectiveness and safety of acupuncture for post-stroke upper limb impairment in multiple aspects and elucidates the underlying neuromuscular effect mechanism of acupuncture to provide clinical evidence.

**Registration:**

International Traditional Medicine Clinical Trials Registry. Registration No. ITMCTR2025000228. November 27, 2024. The study was approved by the Ethics Committee of Guangzhou University of Traditional Chinese Medicine, Shenzhen Hospital (Futian), Ethics. No. GZYLL (KY)-2024–045-01. November 13, 2024.

## 1 Introduction

Ischemic stroke accounts for approximately 60–80% of acute cerebrovascular diseases (1) and is the second leading cause of death and disability worldwide (2). Approximately 55–75% of stroke survivors experience varying degrees of limb dysfunction, with more than 80% affecting the upper limbs (3). Upper limb dysfunction post-stroke is classified as central paralysis, characterized by loss of motor control, including reduced muscle strength and coordination. It is also accompanied by peripheral nerve symptoms such as pain, fine motor dysfunction, and hypoesthesia (4), significantly impacting patients’ quality of life and physical and mental health. How to effectively treat upper limb dysfunction after stroke has become a major clinical challenge in the field of neurorehabilitation (5). The 2021 guidelines from the American Heart Association (AHA/ASA) recommend mandatory exercise therapy (CIMT), task-oriented training, and high-intensity repetitive exercises as first-line treatments for upper limb dysfunction post-stroke (6). However, the effectiveness of these therapies depends on the patient’s voluntary motor ability, and due to a low cost-effectiveness ratio, patient adherence faces significant challenges (7). Furthermore, although therapies such as mirror therapy, occupational therapy, transcranial magnetic stimulation, and upper limb rehabilitation robots are also widely used in clinical practice, their treatment costs are high, and long-term efficacy evidence remains insufficient (8). Therefore, seeking complementary and alternative therapies to facilitate patients’ recovery is essential (9).

Acupuncture is widely recognized and accepted as a complementary and alternative therapy. It involves inserting fine needles into specific acupoints on the skin to achieve “Deqi” and treat diseases. Acupuncture has the unique advantages of good efficacy, ease of operation, and high safety, which have contributed to its increasing influence around the world. According to the World Health Organization (WHO), 11 countries have recognized the clinical efficacy of acupuncture for upper limb dysfunction (10). Moreover, multiple clinical studies have shown that acupuncture treatment has a significant positive effect on upper limb dysfunction after stroke. Compared with rehabilitation therapy alone, acupuncture may enhance upper limb function through potential multi-target neuromodulation mechanisms (11). Furthermore, combining acupuncture with rehabilitation exercises has a synergistic effect, and preliminary research indicates that it can significantly enhance the recovery of muscle spasms, limb movement, and neurological function (12). However, in the existing research on acupuncture treatment for upper limb function after stroke, outcome assessments often rely excessively on subjective scales, leading to questions about the level of evidence for effectiveness in this field. Quantitative outcome assessment tools are needed to further supplement and enhance the credibility of the evidence. Surface electromyography (sEMG) is a technique for recording the bioelectrical signals of the neuromuscular system during muscle activity. It can quantify muscle activation patterns, fatigue levels, and neuromuscular coordination. Due to its specificity and sensitivity to muscle functional status, it has important application value in the quantitative assessment of upper limb functional recovery after stroke (13–15). Crucially, sEMG is easy to operate, non-invasive, and well-accepted by patients (14). However, few acupuncture studies have combined validated subjective assessment tools with sEMG to evaluate the efficacy of acupuncture in treating upper limb dysfunction following a stroke. In addition, the “placebo effect” in acupuncture clinical trials has always been a focus of attention, and there are still doubts about whether sham needle devices can effectively exclude nonspecific effects (16). Some sham acupuncture devices also omit tactile simulation, limiting the effectiveness of blinding in acupuncture trials.

To sum up, this study intends to conduct a single-center, primary investigator-blinded randomized controlled trial (RCT), combining validated scales for assessing upper limb motor function with objective surface electromyography indicators, aiming to evaluate the clinical efficacy of acupuncture treatment for upper limb dysfunction and to explore the neuromuscular driving mechanism of acupuncture in treating upper limb dysfunction after ischemic stroke. At the same time, a novel sham needle device will be used as the control group to provide high-quality evidence-based data to exclude the nonspecific effects of acupuncture.

## 2 Methods

### 2.1 Trial design

This study plans to conduct a single-center, investigator-blinded randomized controlled trial (RCT). By setting up the acupuncture (MA) group and sham acupuncture (SA) group for comparison, it aims to explore the efficacy and safety of acupuncture treatment for upper limb dysfunction in patients with ischemic stroke. Assessment scales related to upper limb function combined with surface electromyography (sEMG) will be used to comprehensively reflect the efficacy of acupuncture and its potential mechanisms. The study will randomly assign patients into two groups at a 1:1 ratio, with 37 cases in each group, recruiting a total of 74 patients.

**Figure 1:**
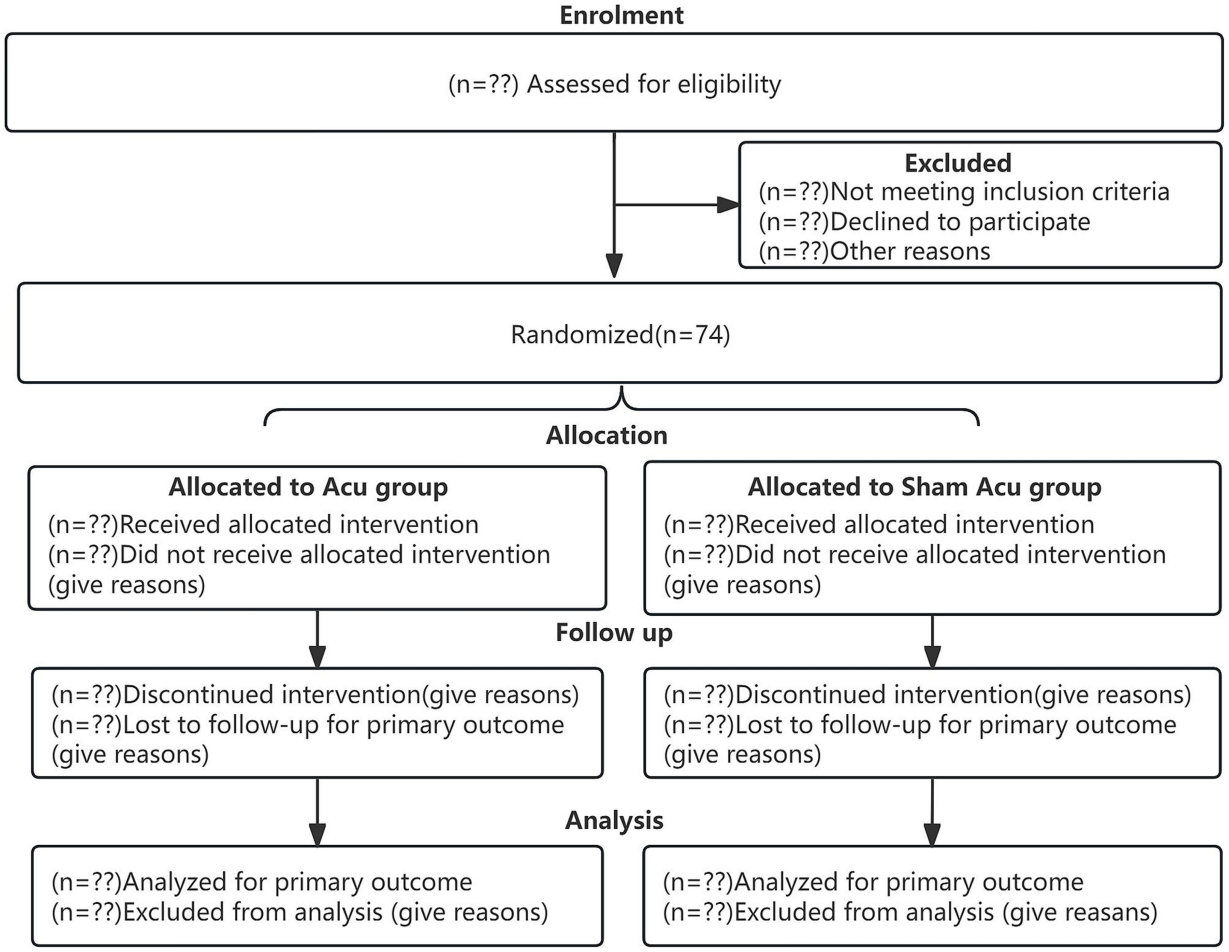
Participants flow chart. CONSORT 2025 Flow charts: Flow charts of the two groups at each stage of the trial (e.g., recruitment, intervention allocation, follow-up, and data analysis). Acu, Manual-Acupuncture; Sham Acu, Sham-Acupuncture.

The subjects will be hospitalized in the Rehabilitation Department of the Guangzhou University of Traditional Chinese Medicine Shenzhen Hospital (Futian). This study has been registered in the international traditional medicine clinical trial, and the details of the trial protocol and statistical analysis plan are available (Registration No. ITMCTR2025000228). This scheme is designed according to the SPIRIT declaration and reported according to the CONSORT 2025 as shown in Figure 1 (17). The schedule of enrolment, interventions, assessments, and visits to the participants is presented in Table 1, which also presents a schematic of the study design.

**Table 1:** Study design.

	STUDY PERIOD						
		Allocation	Treatment				Follow-up
TIMEPOINT**	*-t_1_*	0	*t_1_*	*t_2_*	*t_3_*	*t_4_*	*t_5_*
		Baseline	Week1	Week2	Week3	Week4	3-months
ENROLMENT							
Eligibility screen	X						
Informed consent	X						
NIHSS	X						
Demographic information	X						
Random Allocation		X					
INTERVENTIONS:							
MA			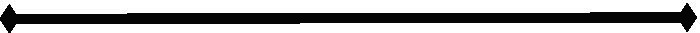				
SA			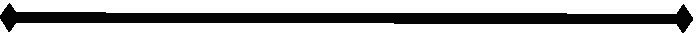				
ASSESSMENTS:							
FMA		X		X		X	X
MSS		X		X		X	X
ARAT		X		X		X	X
MAS		X		X		X	X
SAS		X		X		X	X
SDS		X		X		X	X
sEMG		X				X	
Biochemical indicators		X				X	
Blinding Index		X				X	
Adverse events			X	X	X	X	X

Study schedule of enrollment, interventions, and assessments. The template is from the SPIRIT 2013 statement. HA, hand acupuncture; SA, shame acupuncture; FMA-UE, Fugl-Meyer assessment upper extremity; MSS, Motor Status Scale; ARAT, Action Research Arm Test; MAS, modified Ashworth scale; SAS, Self-Rating Anxiety Scale; SDS, Self-Rating Depression Scale.

### 2.2 Eligibility criteria

#### 2.2.1 Inclusion criteria

The Diagnostic Points of Various Major cerebrovascular diseases in China in 2019 were formulated as per the Branch of Neurology of the Chinese Medical Association (18). The following patients will be included:

(1) They meet the above diagnostic criteria for ischemic stroke.
(2) Aged between 35 and 80 years, sex is not limited.
(3) 2 weeks to 6 months have passed following the first stroke.
(4) Objectively measured upper limb dysfunction; National Institutes of Health Stroke Scale (NIHSS) score ≥ 5.
(5) Conscious and stable vital signs.
(6) Provide signed informed consent.

#### 2.2.2 Exclusion criteria

(1) Transient ischemic attack or reversible neurological impairment (RIND).
(2) Neurological impairment is confirmed to be caused by brain tumors, brain trauma, brain parasitic disease, heart disease, or metabolic disorders, among others.
(3) Severe spasticity of the upper limb.
(4) Pregnant or lactating women.
(5) Combined heart, liver, kidney, hematopoietic system, and endocrine system diseases or other serious primary diseases.
(6) Patients with a history of psychiatric disorders or taking psychotropic drugs.
(7) Other treatments that may influence the results of this study.

#### 2.2.3 Withdrawal criteria

(1) Withdrawal as decided by the investigator:

① During the study, the participant has certain comorbidities, complications, or pathological changes that make it unsuitable to continue the study.
② In the study, the participant has poor compliance, which may affect the evaluation of effectiveness and safety.
③ The participant breaks the blindness or exposes the blinded data in an emergency.
④ Adverse events and serious adverse events occurred, making a participant unsuitable to continue to receive the study.

(2) Withdrawal from the study.

According to the provisions of the informed consent, participants have the right to withdraw from the study midway, or the participants may not explicitly withdraw from the study, but may no longer receive medication and testing, and lose follow-up (which is also classified as a “withdrawal,” called “drop”). The reasons for withdrawal, such as poor efficacy, intolerance to certain adverse effects, inability to continue the clinical study, economic factors, and failure to explain the reasons for the loss of visits, should be understood as much as possible and recorded.

### 2.3 Randomization and allocation concealment

In this study, a centralized randomization system developed by the research team randomly assigned patients to the acupuncture and sham acupuncture groups in a 1:1 ratio. The module size was set to 4, and 37 patients were randomly assigned to each group. All assignment information was unpredictable and invisible until participant inclusion, and the assignment sequences were directly notified to the acupuncturist by the system to ensure fairness of the assignment sequence hiding and randomization process. After the investigator confirms the qualified cases, a third party submits the application to the central randomization system, which automatically assigns the group information according to the randomization method and sends the group information to the investigator and acupuncturist.

### 2.4 Blinding

**Figure 2:**
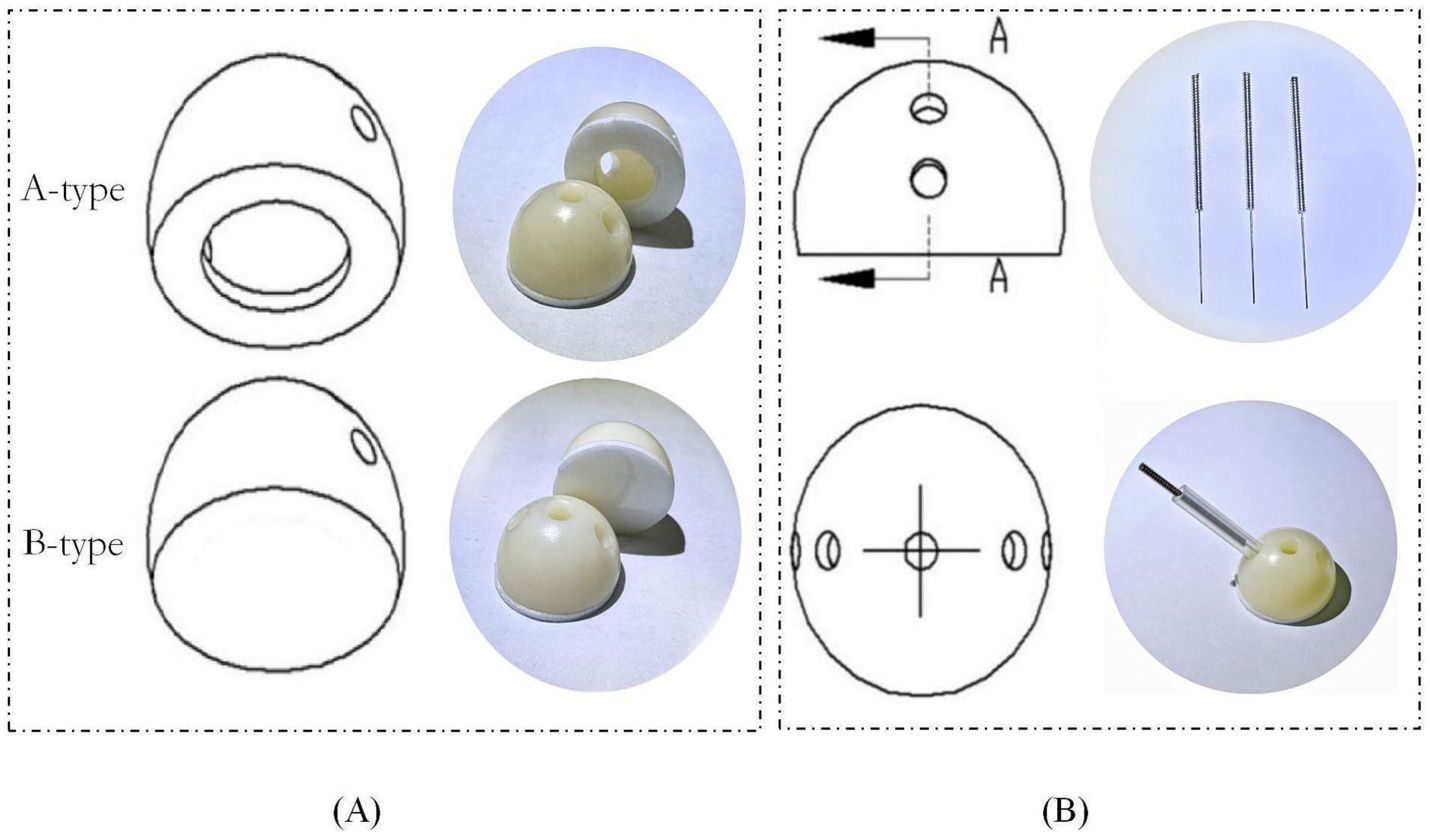
Auxiliary device for acupuncture blind. A-type device is used for MA group with openings in the center. B-type device is used for SA group with no openings. **(A)** is a diagram of the device. **(B)** is a diagram of the device in use. A refers to A-type.

The primary researchers are unaware of the patients’ group assignments. The efficacy evaluation is conducted by third-party personnel, and the database for data statistics is set up with a first-level blind, so the statisticians do not know the specific group details. Due to the limitations of acupuncture procedures, acupuncturists cannot be blinded. They are aware of the patients’ group assignments. All operations are trained in accordance with established Standard Operating Procedures (SOP). The acupuncture blinding auxiliary device was independently developed by the team and has been proven to effectively maintain blinding. According to the requirements, Type A and B blind acupuncture aids will be used in the MA and SA groups, respectively. Auxiliary device for acupuncture blind is presented in Figure 2.

### 2.5 Sample size

The sample size calculation for this study was based on the primary outcome measure, the FMA-UE score. According to preliminary pilot results, the mean difference (MD) in FMA-UE scores between the acupuncture and control group after treatment was 11.5, with a standard deviation (SD) of 10.4. Meanwhile, the minimal clinically important difference (MCID) for the Chinese version of the FMA-UE is 4.6. This study was a superiority design, using PASS 15.0 software for sample size estimation. An independent samples t-test was employed, with a significance level set at *α* = 0.05 and power *β* = 0.8. It is estimated that the acupuncture group must reach the MCID value compared to the sham acupuncture group to be clinically meaningful. Therefore, the superiority margin is set equal to the MCID value of 4.6. The minimum sample size calculated is 58 cases. Considering a 20% dropout rate, the final sample size was determined to be 74 cases (37 cases each in the MA and SA groups).

### 2.6 Interventions and comparison

Acupuncture treatments will be provided by licensed acupuncturists, each with at least 5 years of clinical experience, affiliated with Guangzhou University of Chinese Medicine Shenzhen Hospital (Futian). Acupuncturists will be trained in accordance with established standardized operating procedures (SOPs) to ensure uniformity of acupuncture practice. The content of communication between doctors and patients was also specified to avoid the transmission of group allocation information due to interaction.

#### 2.6.1 Acupuncture intervention

**Figure 3:**
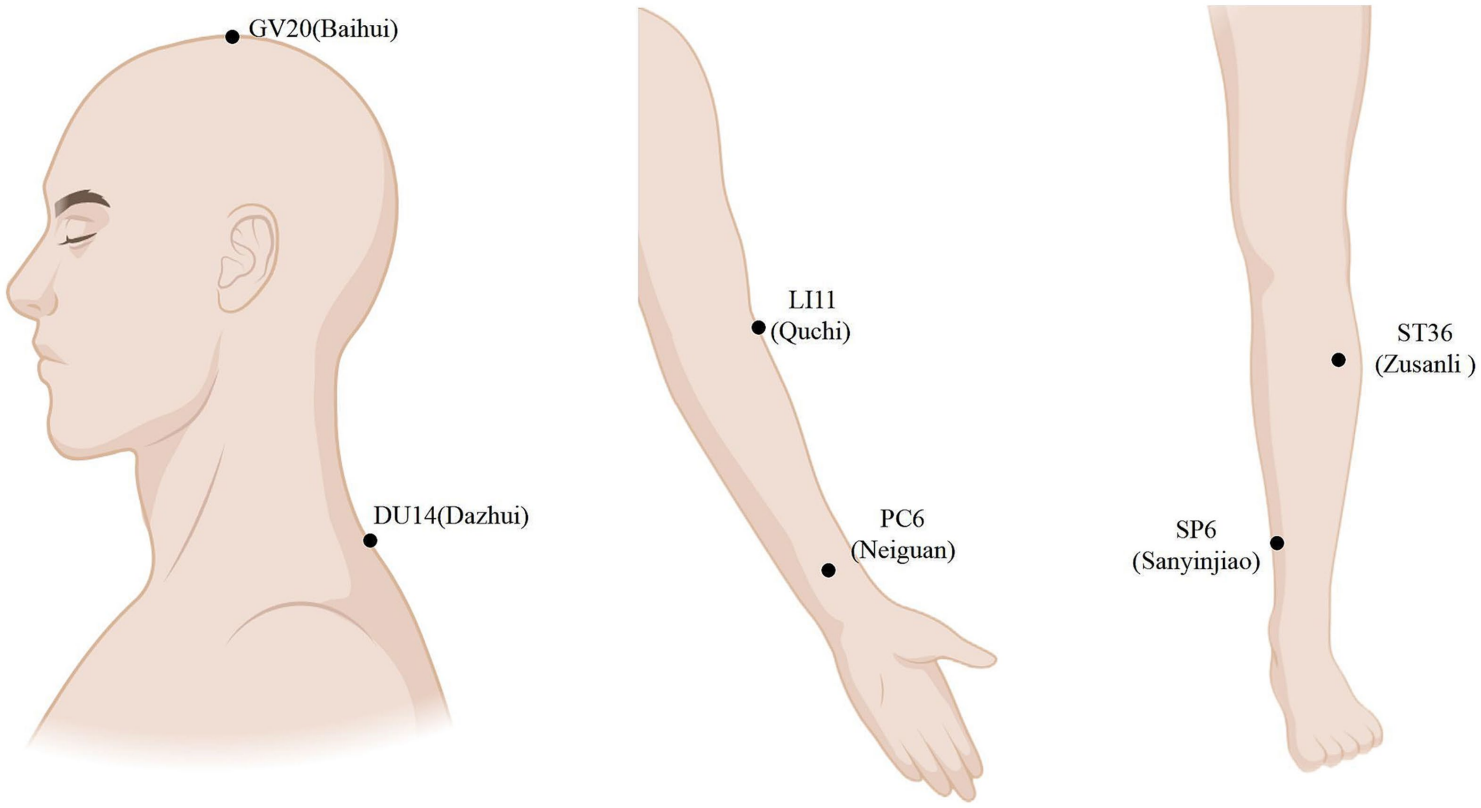
Locations of the MA group.

According to the standardized acupuncture protocol, the MA group uses an A-type acupuncture auxiliary device with a catheter and a hollow base in the middle. Points include bilateral GV20 (Bai hui), DU14 (Da zhui), LI11 (Qu chi), PC6 (Nei guan), ST36 (Zu Sanli), and SP6 (San Yinjiao) (19). A disposable ordinary acupuncture needle (size 0.30 mm × 40 mm or 0.25 mm × 40 mm) is used for the intervention. The needle is inserted along the catheter by thrusting it along the pipe or obliquely for 10–30 mm. The participants are asked about having a sense of distress in identifying the local production of “qi.” Each acupuncture session lasts for 30 min, and the needles are inserted every 15 min, with one session per day, five sessions per week, for 4 weeks. Acupuncture point localization is based on the standards of the World Health Organization Standard Acupuncture Meridian Point Localization (published by People’s Health Press in 2010) (20). The MA group’s point selection and locations are presented in Table 2 and Figure 2 (Figure 3).

**Table 2:** The point selection and location of MA group.

Acupoints	Locations	Depth
GV20 (Bai hui)	At the head, 5 inches above the center of the hairline, or at the midpoint of the line connecting the tips of the ears	0.5–0.8
DU14 (Da zhui)	At the neck, on the posterior midline, in the infraspinal depression of the 7th cervical vertebra	0.5–1.2
LI11 (Qu chi)	At the lateral end of the elbow crease, bend the elbow and reach the midpoint of the line connecting the ulnar and lateral epicondyle of the humerus	0.5–1.2
PC6 (Nei guan)	On the palmar side of the forearm, 2 inches above the wrist crease, between the palmar longus tendon and the radial wrist flexor tendon	0.5–0.8
ST36 (Zu Sanli)	Located on the outer side of the calf, 3 inches below the calf’s nose, on the line connecting the calf’s nose and Jiexi	0.8–1.2
SP6 (SanYinjiao)	3 inches above the tip of the medial malleolus, and the posterior edge of the tibia near the bone edge depression	0.8–1.2

#### 2.6.2 Sham acupuncture intervention

**Figure 4:**
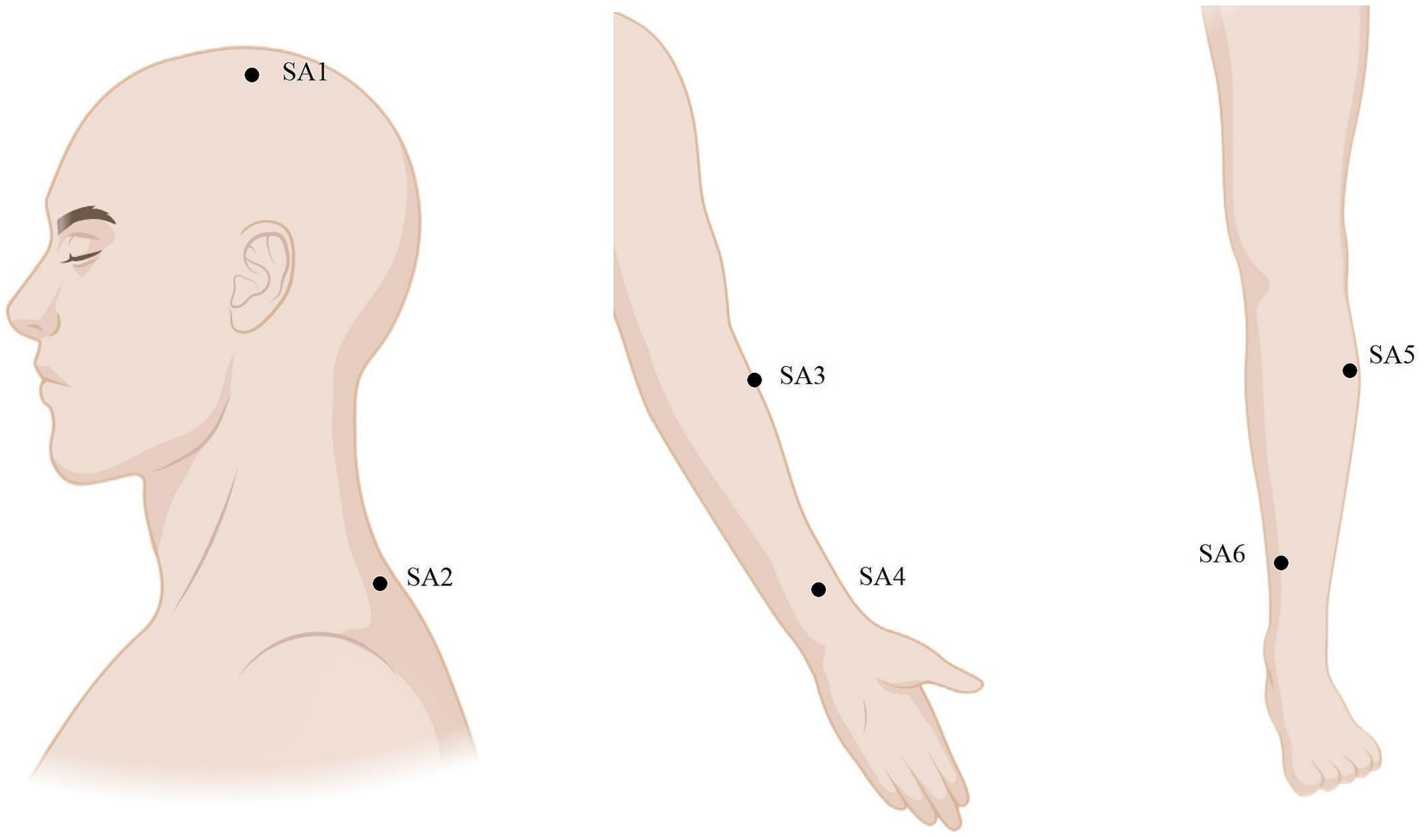
Locations of the SA group.

The SA group follows standardized step-by-step instructions and operations, using a B-type acupuncture auxiliary device equipped with a catheter and a solid base in the middle, and six bilateral non-meridian points with clear localization were selected. The selection and locations of the SA group points are presented in Table 3 and Figure 4. A disposable blunt needle is passed through the plastic catheter on the base and fixed to the skin, applying a certain pressure to simulate the sensation of needling, causing a tingling sensation in the patient without penetration or inducing the sensation of getting “qi.” The treatment protocol is similar to that used in the MA group. The SA group device and operation are shown in Figure 2.

**Table 3:** The point selection and location of SA group.

Sham acupoints	Locations
SA 1	On the head, 5 inches above the hairline, 2 inches apart, and 2 inches apart from the Baihui acupoint
SA 2	At the neck, horizontally level the 7th cervical vertebrae below the spine, and open 1 inch next to the Dazhui acupoint
SA 3	At the outer end of the elbow crease, 1 inch below the Quchi acupoint
SA 4	On the palmar side of the forearm, 2 inches above the wrist crease, and 0.5 inches beside the radial side of the Neiguan acupoint
SA 5	On the outer side of the tibial crest of the calf, 3 inches under the outer knee, and 1 inch outward when the foot is 3 miles apart
SA 6	3 inches straight up from the tip of the medial malleolus, 0.5 inches forward from the Sanyinjiao acupoint on the tibia

#### 2.6.3 Routine treatment

The MA and SA groups receive 4 weeks of routine basic treatment, including nutritional support, and nursing care (including positioning, oropharyngeal care, and pressure ulcer prevention).

### 2.7 Outcomes

At baseline, 2 and 4 weeks post-treatment, and at the 3-month follow-up, several experienced and trained third-party personnel are blinded to the outcome indicators. Simultaneously, sEMG data are collected by a specialized, trained rehabilitation therapist who is not involved in the design, and the RMS, iEMG, and MF of the affected upper limb core muscle group are compared before and after treatment.

#### 2.7.1 Primary outcome

The primary outcome is the Fugl–Meyer Assessment (FMA), which is used in clinical settings and research to assess motor deficits and recovery in individuals post-stroke (21) and is categorized into the FMA-UE (UE, upper extremity) specifically for assessing upper extremity function. The FMA-UE, one of the most widely used and recognized clinical assessment tools for evaluating upper limb motor function, has a total of 33 items and is divided into three scoring grades according to task completion degree (0, 1, and 2 points respectively), with a total score of 66 points; the higher the patient’s score, the better the motor function because of the quantitative indexes, which make its assessment detailed, reliable, and sensitive. According to the study, the change in FMA-UE between the two groups is the minimum clinical difference (MCID), equal to 5.2 (22).

#### 2.7.2 Secondary outcomes

Secondary outcomes include the Motor Status Scale (MSS), Action Research Arm Test (ARAT), Motor Assessment Scale(MAS), self-rated anxiety scale (SAS), and self-rated depression scale (SDS) scores.

The MSS scale is divided into several parts including the shoulder, elbow and forearm, wrist, and hand. It includes five major categories: shoulder movement, elbow and forearm movement, wrist movement, hand movement, and upper limb activities based on hand function, with a total of 29 sub-items and a maximum score of 82. The higher the score, the better the function. MSS is an upper limb motor function assessment method designed by Asien and others based on the upper limb motor section of the Fugl-Meyer Assessment (FMA-UE), addressing the FMA’s lack of evaluation for individual finger motor function. The MSS assessment includes isolated movements that make up complex movements, allowing for a more sensitive reflection of functional impairments and treatment effects (23).

The ARAT scale provides a comprehensive and standardized method to assess upper limb function. It assesses upper limb movement through four basic movements: grasping (six items), griping (four items), pinching (six items), and gross motor (three items). Each item is rated on a 4-point scale of 0 for failure to 3 for normal completion out of 57 points. The evaluation of limb dysfunction caused by cerebral cortical injury involves effectively assessing limited arm movements by evaluating the patient’s ability to manipulate objects of different sizes, weights, and shapes (24).

The MAS score is highly correlated with the FMA and is used to evaluate muscle spasms and muscle tension by observing the degree of muscle tension in different postures to accurately assess motor function (25). The nine items of the scale are scored from 0 to 6 points, in which the total score of the whole-body muscle tension is not included in the total score, and the total score of the eight items is 48 points. Higher scores indicate better motor function. Movement disorders are classified as mild (> 33 points), moderate (17–32 points), or severe (0–16 points).

The SAS is used to assess the subjective feelings of patients with anxiety. It is a 4-point self-rating scale containing 20 items. Higher scores indicate more severe symptoms (26).

The SDS is a tool for individual self-assessment of depressive symptoms (27). It contains 20 items divided into positive scores (1–4 points) and reverse scores (4–1 points). The cutoff value of the standard scores is 53 points; 52–62 is classified as mild depression, 63–72 as moderate depression, and >72 as severe depression.

#### 2.7.3 Other outcomes

sEMG is a technique for recording the bioelectrical signals of the neuromuscular system during muscle activity (13), which can quantify muscle functional status. It was recorded using a 10-channel multi-lead 060525001 Myotrac3 surface electromyograph (THOUGHT, Canada). The evoked potentials of the core upper limb muscle groups (including pectoralis major, deltoid, biceps brachii, triceps brachii, brachioradialis, etc.) defined by neuroanatomy experts were collected. The specific indicators include the root-mean-square value (RMS) and integrated electromyography value (iEMG) of the time-domain indicators, which quantify the overall muscle activation strength from the perspectives of transient intensity and cumulative loading, respectively. Frequency domain indicators include the median frequency (MF), defined as the frequency point corresponding to 50% of the energy accumulation in the spectrum, to infer muscle fiber conduction velocity (MFCV), which is used to assess muscle fatigue (28). Before collection, evaluators need to undergo standardized training to regulate the movement patterns of the core muscle groups; then the evaluators guide the patients in exercise to practice and master the standardized movement methods; secondly, before collection, the signal acquisition parameters of the instrument need to be pre-tested to ensure parameter stability. The specific operation is as follows: Inform the patient before the test to perform relevant actions according to sEMG. The patient is in a sitting position before applying the electrode sheet, local hair removal is carried out on the site where the electrode sheet is to be applied, the skin surface is polished with a special sanding pad, and then wiped with an ethanol cotton ball to remove the skin cuticle, reduce the contact resistance between the electrode and the detection surface, and protect the electrode to avoid movement and avoid errors as much as possible. After completing the electrode affixing, the electromyographic signal becomes stable, and detection begins. According to the machine’s instructions, the doctor and patient cooperate to complete the corresponding active and passive movements. Each movement lasts for 15 s, followed by a 5-s rest. Each movement is repeated 3 times as one set, with a 1-min rest after each set. The middle 10 s of the electromyographic signal for each movement is used for analysis.

#### 2.7.4 Blinded index

Participants were asked to guess the type of acupuncture they received at the end of the acupuncture treatment. A third party recorded the data, and statisticians used the SAS macro %BBI2 for visual analysis to assess the success rate of blinding in the randomized controlled trial (29). The environment for this macro is based on SAS V.9.4 (SAS Institute, Cary, North Carolina, United States). For each group (e.g., MA and SA), the BBI is calculated as:


$${\mathrm{BBI}}_{n}=(2•T_{n}^{2}-1)•(\frac{N_{n,\text{correct}}+N_{n,\text{wrong}}}{N_{n,\text{total}}})$$

$$T_{n}^{2}=\frac{N_{n,\text{correct}}}{N_{n,\text{correct}}+N_{n,\text{wrong}}}$$

$$N_{n,\text{total}}=N_{n,\text{correct}}+N_{n,\text{wrong}}+N_{n,\text{unknown}.}$$

BBI ∈ [−1, 1], with 0 indicating a random guess; BBI ∈ [−0.2, 0.2] indicates a successful blinding.

### 2.8 Adverse events and safety

#### 2.8.1 Definition and handling of adverse events

Any incidents, symptoms, signs, or abnormal examination results occurring between the intervention and the last follow-up. Common needle-related adverse events (AE) include broken needles, dizziness, and infection. Serious adverse events (SAE) refer to any experience or condition that poses significant harm to the patient or contraindicates continued treatment, such as stroke recurrence or organ damage. During the research process, as soon as a patient expresses discomfort, the physician will immediately stop treatment assess the patient’s signs, symptoms, type, and extent of discomfort, and take appropriate measures to protect the safety of the subject. The patient will be followed up until the condition stabilizes after the adverse event occurs.

#### 2.8.2 Recording and reporting of adverse events

Any AE was recorded in the CRF by the investigator within 24 h. AEs, SAEs, and unanticipated events will be recorded following reporting standards and reported to the Ethics Committee and other regulatory authorities. The principal investigator will regularly review all adverse events, and the Ethics Committee will also receive interim results. If necessary, meetings will be held to reassess the benefits and risks of the trial.

#### 2.8.3 Security analysis

AEs are classified and reported according to common AE evaluation criteria.

Incidence = (number of patients with AE/total number of patients) × 100%

Fisher’s exact test is used to compare intergroup incidence.

Individual list description risk ratio (RR), and confidence interval (CI) calculations are performed for SAEs. Comprehensive recording and analysis ensure subject safety and data integrity, in order to complete statistical analysis and help investigators understand the potential effects of acupuncture.

### 2.9 Data collection and management

The experimental protocol was monitored and revised by acupuncturists, physicians, researchers, and statisticians. Additionally, quality control measures such as informed consent, subject screening, intervention, statistical analysis, and data management are reviewed. In addition, there is regular supervision by clinical experts. This study uses the Case Report Form (CRF) to collect data. Manuscript information and a web database of all study results will be recorded, and information in the repository will be made available after the study is completed.

### 2.10 Data processing and statistical analysis

Data analysis was performed by qualified personnel not involved in the study using the Modified Intention-to-treat (mITT) principle. mITT was defined as participants who were randomized, received at least one treatment, and were statistically analyzed using the SPSS (IBM SPSS Statistics, Version 26.0) software. The significance level was set at 5%. Continuous variables that conform to the normal distribution are represented by mean ± standard deviation (x ± s), and non-normal continuous variables are represented by median and quartile [M (P25, P75)]. Categorical variables are expressed as frequency (percentage) using a chi-square or Fisher precision test. For primary and secondary outcomes, an independent sample T-test was used to compare groups if normal distribution and homogeneity of variance were met; otherwise, the Mann–Whitney *U* test was used. Intragroup comparisons were performed using the paired sample T-test. According to missing outcome data, the last observation carried forward (LOCF) method or multiple interpolation was used to fill in the missing values. In addition, sensitivity analyses were performed to clarify the robustness of the results by comparing the conforming protocol analysis set (PPS) analysis with the ITT analysis set. The data obtained by sEMG were analyzed with the main outcome, FMA-UE, using Person/Spearman correlation analysis. Finally, we calculated the BI index to evaluate the success of blinded implementation.

## 3 Discussion

This study aims to conduct a prospective, principal investigator-blinded, randomized controlled trial of efficacy, innovatively combining subjective scales (such as FMA-UE and ARAT) with objective indicators (sEMG). The goal is to evaluate the efficacy and safety of acupuncture in treating post-stroke upper limb dysfunction and to further enhance the credibility of the evidence.

As one of the most popular complementary and alternative therapies worldwide, acupuncture has received widespread attention in the field of post-stroke motor function. At the evidence-based level, although numerous clinical studies have demonstrated the effectiveness of acupuncture, the non-specific “placebo effect” and the inherent therapeutic effects of acupuncture have not been fully distinguished, leading to ongoing skepticism about its efficacy. Sham acupuncture, which closely resembles real acupuncture in appearance and patient perception but only simulates the sensation of needle insertion, has become the primary method in recent years to minimize the “placebo effect” of acupuncture. It is worth noting that there is still considerable controversy regarding the ability of sham acupuncture to completely eliminate the placebo effect; however, it remains the best available solution at present. Additionally, we have observed that current placebo needles have the flaw of not being able to achieve completely parallel needle insertion. Based on this, the research team has improved and developed a placebo needle device capable of fully parallel insertion and achieving complete blinding of subjects in terms of appearance and insertion sensation, to control for non-specific factors related to acupuncture during the study and to minimize the potential placebo effect of acupuncture (30), avoiding overestimation or underestimation of the efficacy of acupuncture, thus improving the quality of the study and the reliability of the results. At the level of mechanism of effect, existing studies have reported that acupuncture enhances muscle control, including muscle strength and limb coordination (31), improve muscle atrophy condition after stroke (32), to promote the recovery of limb dysfunction related to stroke. Our preliminary research also reported that acupuncture can restore neural dysfunction and improve post-stroke upper limb motor impairment by regulating the activity of pyramidal neurons and parvalbumin (PV) neurons, warranting further investigation to uncover the precise mechanisms of acupuncture’s effects (33).

However, due to the current lack of a sound safety system for acupuncture adverse events (AEs), concerns about the safety of acupuncture have arisen, which to some extent has limited the clinical promotion of acupuncture. Therefore, safety should be considered a prerequisite for efficacy research during acupuncture treatment. The occurrence of AEs is closely related to acupuncture operations. We have developed a well-structured standardized operating procedure (SOP) related to acupuncture treatment based on operation-related factors and strengthened effective communication among doctors during treatment to minimize the incidence of AEs (34). However, AEs cannot be completely avoided at present. Identifying the causes of AEs and fully reporting them can provide valuable data support for avoiding similar incidents in the future. Thus, we use a unified reporting standard to timely and objectively record AEs in research, providing experiential support to improve acupuncture safety. Enhancing safety increases the persuasiveness of acupuncture efficacy, promotes the clinical application of acupuncture for post-stroke upper limb dysfunction, and comprehensive scientific evaluation will also help improve the quality of case study reporting.

In addition, the efficacy evaluation of acupuncture treatment for post-stroke upper limb dysfunction currently mostly uses scales. Although the sensitivity and specificity of subjective assessment tools have improved compared to before (35), they still cannot avoid interference from subjective factors of evaluators and patients, which may ultimately lead to bias in acupuncture efficacy. As a tool reflecting effect mechanisms, sEMG has been widely used in recent years for the objective assessment of post-stroke limb function. By recording muscle activity information during movement or rest (such as contraction timing, location, and intensity) and performing time-frequency analysis to quantify peripheral bioelectrical parameters like muscle co-contraction rate and motor unit recruitment timing, it visualizes muscle electrical activity and evaluates muscle activation patterns (36). However, there are currently few RCT studies combined with sEMG. Therefore, this study innovatively integrates sEMG and related scales, using both subjective and objective indicators to conduct a multidimensional assessment of acupuncture’s improvement on post-stroke upper limb function. The aim is to overcome the limitations of single efficacy evaluation and weak mechanism explanation, achieving dynamic and visualized analysis of the acupuncture effect mechanism. In addition, this study also conducts correlation analysis between the scales and sEMG indicators, intending to establish a comprehensive evaluation system of the “functional recovery-muscle synergy” full chain, further exploring the neuromuscular driving mechanism.

However, the clinical application of sEMG still faces a major challenge: its data collection is easily affected by various confounding factors such as the environment, leading to inaccurate evaluation of sEMG efficacy. Therefore, to control the quality of data collection, we specify the muscle movement paradigm before collection, uniformly treat the subject’s skin tissue, use standardized electrodes and instruments during collection, isolate the patient from environmental noise, and improve the accuracy of results by controlling the influence of these related operational factors.

In summary, this study will use a randomized single-blind trial combining subjective and objective indicators to evaluate the efficacy and safety of acupuncture in treating upper limb dysfunction and explore its neuromuscular mechanisms. It aims to provide intuitive and sensitive responses to the therapeutic effects of acupuncture rehabilitation, further supporting data for precision rehabilitation.

## 4 Limitations

Owing to the specificity of acupuncture clinical research, this study is not blinded to the acupuncturists, potentially introducing an operational bias. In addition, the small sample size and single-center design limit the external extensibility of the study, and the 3-month follow-up period is also insufficient to assess long-term efficacy. To determine the effectiveness of acupuncture, the research team intends to conduct a multicenter randomized controlled trial, including larger sample size, extend the follow-up to 12 months, and combine functional magnetic resonance imaging and metabolomics technology to further explore the mechanism of action of acupuncture in the treatment of post-stroke upper limb dysfunction.
